# Arab world’s impact on bladder cancer research and opportunities for growth: A bibliometric review study

**DOI:** 10.1097/MD.0000000000037554

**Published:** 2024-03-22

**Authors:** Mustafa Saleh, Peter Raffoul, Alvar Akil, Paul Bassil, Pascale Salameh

**Affiliations:** a Faculty of Medical Sciences, Lebanese University, Beirut, Lebanon; b School of Medicine, Lebanese American University, Byblos, Lebanon; c Institut National de Santé Publique, Epidémiologie Clinique et Toxicologie (INSPECT-LB), Beirut, Lebanon; d University of Nicosia Medical School, Nicosia, Cyprus.

**Keywords:** Arab world, bibliometrics, bladder cancer, Embase, PubMed, urology

## Abstract

**Background:**

Bladder Cancer (BC) is a widespread form of cancer that affects over 1.6 million people globally. The majority of cases are diagnosed as urothelial carcinoma, with a higher likelihood of diagnosis in men and with increasing age. The Arab world (AW) is one of the regions with the highest incidence and mortality rates of BC, and the average age of diagnosis is between 40 and 49 years in North Africa and the Middle East. This study aims to assess the activity and distribution of BC publications in the AW.

**Methods:**

A systematic search across MEDLINE and Embase databases spanning 2007 to 2021 identified 1208 English-language articles on bladder cancer with Arab affiliations. The dataset was normalized against the average population and GDP (2007–2020) for 22 Arab countries. Statistical analyses via SPSS and visualizations with VOSviewer unveiled collaboration patterns and thematic trends in Arab bladder cancer research.

**Results:**

A total of 1208 BC publications were published in the AW, representing 0.24% of all biomedical publications. Egypt topped the list with the highest number of publications. The co-authorship analysis generated by VOSviewer revealed that out of 4766 authors, 161 met the minimum threshold of 5 publications.

**Conclusion:**

The findings reveal that Egypt and Jordan are at the forefront of BC research in the region, while other Arab countries are lagging behind despite being heavily impacted by the disease. To drive progress in the field, it’s important to uncover the obstacles impeding BC research in these countries and implement effective solutions to overcome them.

## 1. Introduction

Bladder Cancer (BC) is a complex form of cancer with diverse histological characteristics and several associated risk factors. The majority of BC cases are diagnosed as urothelial carcinoma (75%), with the remaining 25% being attributed to variant histologies.^[1]^ BC makes up 90% to 95% of all urothelial carcinoma cases.^[2]^ It groups non-muscle invasive bladder cancer (NMIBC) and muscle invasive bladder cancer (MIBC). NMIBC encompasses pathological stages below T2, while MIBC encompasses stages T2 and above.^[3]^ BC is considered a widespread malignancy, impacting a significant number of individuals worldwide, with over 1.6 million people living with the condition.^[4]^ The likelihood of developing BC is higher in men (1.1%) compared to women (0.27%), and tends to increase with age.^[5]^ Risk factors for BC include exposure to carcinogens such as tobacco smoke and occupational chemicals, chronic inflammatory conditions, and radiation therapy. There is a strong connection between smoking and BC, with an estimated 50% population-attributable risk.^[6]^

The Arab world (AW), recording some of the highest incidence and mortality rates in BC, consists of 22 countries spanning Northern Africa with 10 countries and Western Asia with 12 countries. Its population is estimated at around 360 million people.^[7,8]^ While BC is mostly prevalent in Northern America and Europe, countries like Syria and Egypt record comparable age-standardized incidence rates (ASR).^[9]^ Lebanon holds one of the highest ASRs in males among the world population for BC, with a rate of 34.0, surpassing other countries that have previously been identified as having high ASR values, such as Belgium and its neighboring nation, Egypt, which have ASR values of 32.0 and 26.3, respectively.^[10,11]^ Egypt reports the highest mortality rate for BC globally.^[9]^ The Western Asia and Northern Africa regions, which largely encompass Arab countries, collectively exhibit a high region-specific incidence of BC in both genders, indicative of a complex etiology with interrelated risk factors.^[10,12,13]^

The underlying causes of BC are complex and influenced by both modifiable and non-modifiable risk factors. Advanced age is a prominent non-modifiable risk factor, with the average age of diagnosis ranging from 70 to 84 years in developed countries. On the other hand, the average age of diagnosis in North Africa and the Middle East, where schistosomiasis is prevalent, is between 40 and 49 years.^[10]^ The protozoan infection schistosomiasis, caused by schistosoma haematobium, has been identified as a significant risk factor for BC, contributing to its high incidence and mortality in some regions, particularly North Africa.^[12,14]^ In addition, the increasing trend of cigarette and shisha smoking in the AW has been identified as a major modifiable risk factor for BC.^[15,16]^

Given the significance of BC as a health issue in the AW, it is imperative to closely examine the activity of studies published on BC in the AW. Therefore, this study aims to comprehensively assess the AW impact on BC, specifically by quantifying research activity, highlighting potential research gaps, and shedding light on initiatives and strategies aimed at addressing these gaps.

## 2. Methods

### 2.1. Search strategy

A search of the MEDLINE (through PubMed) and Embase databases was conducted to retrieve articles related to BC with an Arab affiliation published in the last 15 years.

In PubMed, MeSH (medical subject headings) terms, author affiliation, and Boolean operators (AND, OR, and NOT) were used for this purpose. A similar method was used in Embase. The keywords employed were “bladder cancer,” “bladder neoplasm,” “bladder carcinoma” and “bladder malignancy.”

The search included all articles related to BC written in English and published between 2007 and 2021 inclusive with at least one author affiliated to an institution located in any of the 22 Arab countries: Algeria, Bahrain, Comoros, Djibouti, Egypt, Iraq, Jordan, Kuwait, Lebanon, Libya, Mauritania, Morocco, Oman, Palestine (Gaza and West Bank), Qatar, Saudi Arabia (KSA), Somalia, Sudan, Syria, Tunisia, the United Arab Emirates (UAE), and Yemen. This systematic search process resulted in a total of 1371 articles.

Articles with authors affiliated with institutions in Lebanon counties in the US and Front Lebanon in the UK were excluded. Similarly, articles with authors affiliated with the Radio-Oncology Centre KSA-KSB in Switzerland, confused with KSA (Kingdom of Saudi Arabia) were excluded. Articles with authors named Jordan were also excluded.

This review is exempt from Institutional Review Board approval, as it involves the synthesis and analysis of existing literature without the collection, analysis, or reporting of original data involving human subjects.

### 2.2. Screening and selection process

Publications were screened for duplicates. A manual screening was further done to identify any undetected duplicates and ensure that all publications were relevant to BC. The screening was done by two reviewers and conflicts were resolved by a third reviewer. Following this comprehensive screening process, the number of articles meeting the inclusion criteria was reduced to 1208.

To address biases among different Arab countries, the average population and gross domestic product (GDP) for the time period 2007 to 2020 were computed for each country using data retrieved from the World Bank.^[17]^ The number of publications obtained after the screening was then normalized against the average population and GDP.

### 2.3. Statistical analysis

The data was then imported to SPSS version 26.0 and Spearman correlation was used to evaluate the relationship between the number of publications and the average population and GDP. The visualization software VOSviewer version 1.6.19 was used to identify the most prominent authors and most common keywords and generate networks illustrating the patterns of collaboration among Arab authors as well as the trends in the keywords used in Arab BC publications.

## 3. Results

**Figure 1.**
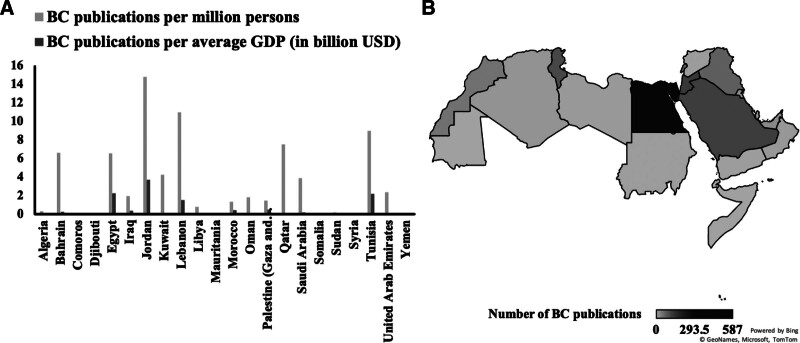
(A) Distribution of Bladder Cancer publications per million persons and GDP per country. (B) Bladder Cancer research activity in the Arab World. GDP = gross domestic product.

A total of 1208 BC publications were published, among the 22 Arab countries analyzed, representing 0.24% of all Arab biomedical publications (Table 1). Egypt topped the list with the highest number of BC publications at 587 (Fig. 1B). Meanwhile, Jordan had the highest proportion of BC publications with 0.44% of the country’s biomedical publications being related to BC. The 22 countries had an average population of 383 million, with Egypt recording the largest population at 90 million and Comoros the smallest at 1 million. The average GDP of the 22 countries was 2.471 billion USD, with the highest GDP recorded in the Kingdom of Saudi Arabia (KSA) at 648 billion USD and the lowest in Comoros at 1 billion USD. On a per-country basis, Egypt, Jordan, and KSA ranked first, second, and third in terms of number of BC publications, with 587, 126, and 117 publications respectively. On the other end of the spectrum, Somalia, Mauritania, Djibouti, and Comoros all ranked last with zero BC publications.

**Table 1:** Number of BC publications in the last 15 years and average population estimates and GDP per country.

Country	BC Publications	Non-BC Publications	All biomedical publications	Proportion of BC publications (%)	Average Population (in million)	Average GDP (in billion)
Egypt	587	137,547	138,134	0.42	90	259
Jordan	126	28,491	28,617	0.44	9	34
KSA	117	130,101	130,218	0.09	30	648
Tunisia	99	34,771	34,870	0.28	11	45
Iraq	66	28,322	28,388	0.23	34	178
Lebanon	65	18,413	18,478	0.35	6	43
Morocco	46	21,146	21,192	0.22	34	103
UAE	21	26,952	26,973	0.08	9	352
Qatar	17	18,859	18,876	0.09	2	154
Kuwait	15	11,816	11,831	0.13	4	134
Algeria	12	13,423	13,435	0.09	39	173
Bahrain	9	4423	4432	0.20	1	31
Oman	7	10,673	10,680	0.07	4	75
Palestine (Gaza & West Bank)	6	5034	5040	0.12	4	13
Libya	5	1510	1515	0.33	6	53
Syria	4	2171	2175	0.18	19	86
Sudan	4	7509	7513	0.05	38	45
Yemen	2	2961	2963	0.07	26	32
Somalia	0	334	334	0	13	6
Mauritania	0	264	264	0	4	6
Djibouti	0	179	179	0	1	2
Comoros	0	80	80	0	1	1
Total	1208	504,979	506,187	0.24	383	2471

BC = bladder cancer, GDP = gross domestic product, KSA = Kingdom of Saudi Arabia, UAE = United Arab Emirates.

Adjusting BC publications per million persons and per GDP for each country resulted in varying country rankings (Table 2). Jordan emerged as the leader when considering BC publications per million persons, with 14.82 publications, followed by Lebanon and Tunisia with 10.99 and 8.97 publications respectively. However, when considering BC publications per GDP, Jordan ranked first, Egypt second, and Tunisia third, with 3.71, 2.27, and 2.21 publications per billion USD, respectively. These results were adjusted to eliminate any biases that may have arisen from the largely variable population size and GDP of the different Arab countries (Fig. 1A).

**Table 2:** BC publications per million persons and per GDP with their respective adjusted ranks.

Country	BC publications per million persons	Rank adjusted for population	BC publications per GDP (in billion USD)	Rank adjusted for GDP
Egypt	6.54	6	2.27	2
Jordan	14.82	1	3.71	1
KSA	3.87	8	0.18	9
Tunisia	8.97	3	2.21	3
Iraq	1.95	10	0.37	7
Lebanon	10.99	2	1.52	4
Morocco	1.35	13	0.45	6
UAE	2.38	9	0.06	17
Qatar	7.52	4	0.11	11
Kuwait	4.27	7	0.11	10
Algeria	0.31	15	0.07	15
Bahrain	6.63	5	0.29	8
Oman	1.81	11	0.09	13
Palestine (Gaza & West Bank)	1.45	12	0.48	5
Libya	0.78	14	0.09	12
Sudan	0.21	16	0.05	18
Syria	0.11	17	0.09	14
Yemen	0.08	18	0.06	16
Comoros	0	19	0	19
Djibouti	0	19	0	19
Mauritania	0	19	0	19
Somalia	0	19	0	19

BC = bladder cancer, GDP = gross domestic product, KSA = Kingdom of Saudi Arabia, UAE = United Arab Emirates.

After carrying out the data analysis, a significant moderate positive correlation between the number of BC publications and average GDP (*r* = 0.68, *P* = .001) was found. Additionally, a low positive correlation was found between the number of BC publications and average population (*r* = 0.384, *P* = .078), although this relationship was not statistically significant.

**Figure 2.**
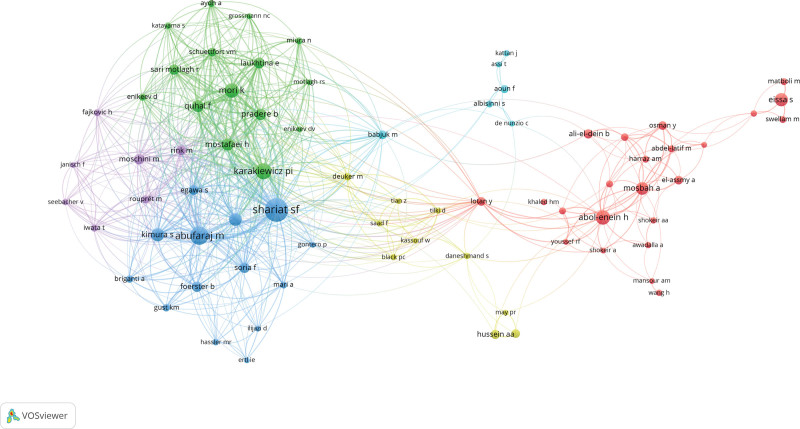
Co-authorship cluster map, with the size of the circles being directly proportional to the number of publications produced by the author, and the thickness of the curve being directly proportional to the number of collaborations.

**Figure 3.**
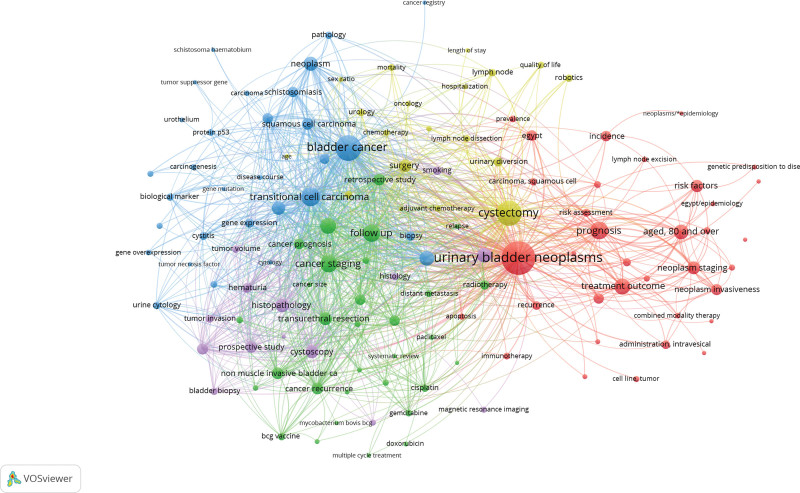
Keyword co-occurrence cluster map, with the size of the circles being directly proportional to the keyword occurrence and the thickness of the curve being directly proportional to keywords co-occurrence.

The co-authorship analysis generated by VOSviewer revealed that out of 4766 authors, 161 met the minimum threshold of 5 publications. Among the 161 authors, the largest set of collaborating coauthors consisted of 75 authors in total and is illustrated by the cluster map in Figure 2. Shariat SF, Abufaraj M, and Karakiewicz PI had the highest number of publications with 83, 51, and 39 publications, respectively. While Shariat SF and Abufaraj M had the highest number of collaborations among Arab authors, fewer substantial collaborations were noted between other authors. The keyword co-occurrence analysis resulted in 6043 keywords with the top 5 keywords being “urinary bladder neoplasms,” “bladder cancer,” “cystectomy,” “transitional cell carcinoma,” and “prognosis” (575, 343, 321, 183, and 160 occurrences, respectively). Figure 3 illustrates the co-occurrence of the most common keywords grouped into 5 clusters. Of the keywords, “Egypt” and “schistosomiasis” were remarkable with 60 and 55 occurrences, respectively.

## 4. Discussion

Our study analyzed the number of BC publications in 22 Arab countries and found that Egypt had the highest number of BC publications with 587 publications in 2021, while Jordan had the highest proportion of BC publications per biomedical publication. When considering BC publications per million persons, Jordan emerged as the leader, followed by Lebanon and Tunisia. However, when considering BC publications per GDP, Jordan ranked first, Egypt second, and Tunisia third. The study found a moderate positive correlation between the number of BC publications and average GDP, and a low positive correlation between the number of BC publications and average population, although this relationship was not statistically significant.

To date, a thorough global bibliometric analysis encompassing both NMIBC and MIBC has yet to be conducted. Although a bibliometric analysis was carried out between the years 2001 and 2022, it was limited to NMIBC only and found 2185 articles globally, with the United States, China, and Italy ranking first, second, and third, respectively, with 559, 315, and 262 articles.^[18]^ Our results show that the top 3 Arab countries in terms of the number of BC publications (Egypt, Jordan, and KSA) have comparable numbers to the top three countries listed in the previous study. This indicates that a part of the AW is actively addressing the issue of BC and efforts are being made to progress in this field, given the high incidence and mortality ASRs in the region, which is not expected to significantly decrease in the near term.^[19]^ Additionally, our study showed that Egypt topped the list in terms of number of BC publications and was a prominent keyword along with the keyword “schistosomiasis,” a proven risk factor for BC and an endemic disease in Egypt. This reflects the country’s efforts to further investigate BC and its risk factors given that it has the highest mortality rate for BC globally.

Our study revealed a significant correlation between GDP and the number of BC publications, therefore suggesting that economic factors may play a role in the production of publications.^[20]^ A recent worldwide bibliometric analysis of BC between 2000 and 2020 by Awada et al, utilizing a method strategy comparable to ours, revealed a significant correlation between GDP and research output, which is in line with the findings of our study.^[21]^ This can be seen in Lebanon where the negative effects of the 2019 port explosion exacerbated by the ongoing economic recession have likely reduced the country’s ability to produce publications.^[22,23]^ The financial instability in Lebanon has also affected its ability to support important resources such as trained healthcare professionals and funding for clinical research.^[24]^ This issue extends beyond Lebanon and affects the whole Arab region, which continues to face inter- and intra-regional conflicts, economic instability, and political corruption, all of which have an adverse impact on the research.^[25]^

The Arab region is anticipated to experience a rise in smoking prevalence and tobacco-related health problems due to the lack of stringent surveillance systems and anti-tobacco policies.^[16]^ This is exemplified by the growing smoking trend among university students in the AW.^[15]^ Despite that, our study demonstrates that several Arab countries produce minimal BC publications and continue to lag in addressing the damaging effects of having one of the highest smoking rates globally.^[26]^ Moreover, Arab authors’ collaborations seem to be weak prompting the need for joining efforts to address the concerning BC rates in the AW.

The underrepresentation of BC publications in the AW can be attributed to several factors, including the limited focus on clinical research in tertiary care hospitals and physicians focusing on patient care only with limited biomolecular and clinical research in the region.^[27]^ The region also lacks a research culture, with its physicians being mostly clinically trained rather than academically equipped to produce, review, and reproduce research fields.^[25]^ In addition, medical students lack a research-oriented mindset due to a lack of emphasis on research in the educational system and inadequate encouragement from universities.^[28]^ Furthermore, there is a widespread shortage of funding for basic, translational, and clinical research in the region, except for a few countries in the Gulf Cooperation Council, which can be attributed to economic instability, political disorder, and untrained healthcare professionals who are unable to secure funding.^[29]^ Finally, the absence of research facilities and equipment, as well as the lack of international or regional partnerships or joint PhD programs, contributes to the underrepresentation of high-quality research in the AW.^[25,30]^

Potential areas of research in BC are numerous as the field is evolving towards personalized medicine, with a heightened emphasis on the genetic and biomarker components of the disease. These developments, in conjunction with advancements in surgical and intravesical treatment methods, are expected to lead to improved patient outcomes.^[31]^ Through the use of genomics, the classification of the disease pathology has been improved, resulting in pathology-specific management strategies and more accurate prognostic evaluations.^[32,33]^ However, the identification of genetic and other biomarkers that would allow for tailored management is still an area of ongoing research.^[34]^ Despite this progress, screening and palliative care have received limited attention and remain under-investigated, although they are crucial for patients with advanced disease and poor prognosis.^[35]^

There are several limitations to this study. First, the study only considered articles published in English and with authors affiliated with institutions located in 22 Arab countries. This means that the study may have missed articles published in other languages or by authors affiliated with institutions outside of the specified countries. Second, the use of World Bank data to compute the average population and GDP for each country over the specified time period may not accurately reflect the actual population and GDP for each country during this time. Third, the search strategy employed may have overlooked articles that could have been retrieved using alternative keywords. Finally, the reliance on only Embase and PubMed databases for data collection could have led to the exclusion of articles indexed in other databases.

## 5. Conclusion

To summarize, our study analyzed the BC publication output in 22 Arab nations and discovered that Egypt produced the highest amount with 587 publications, while Jordan had the highest proportion of BC publications per biomedical publication. This study provides insight into the current state of BC research in the AW and highlights potential areas for growth. This research is unique in its thorough examination of BC publications in the region and identifies potential areas for improvement. The AW has the capability to significantly impact the global efforts against BC through the promotion of collaboration, partnerships, and investment in BC research.

## Author contributions

**Conceptualization:** Mustafa Saleh, Alvar Akil, Peter Raffoul, Paul Bassil, Pascale Salameh.

**Data curation:** Mustafa Saleh, Alvar Akil, Peter Raffoul, Paul Bassil.

**Formal analysis:** Mustafa Saleh, Alvar Akil, Peter Raffoul, Paul Bassil.

**Methodology:** Mustafa Saleh, Alvar Akil, Peter Raffoul, Paul Bassil, Pascale Salameh.

**Writing – original draft:** Mustafa Saleh, Alvar Akil, Peter Raffoul, Paul Bassil.

**Writing – review & editing:** Mustafa Saleh, Peter Raffoul, Pascale Salameh.
